# Impact of COVID-19 pandemic on the course of refractory chronic spontaneous urticaria under omalizumab treatment^☆^

**DOI:** 10.1016/j.abd.2022.08.006

**Published:** 2022-12-14

**Authors:** Müge Olgaç, Osman Ozan Yeğit, Şengül Beyaz, Nida Öztop, Can Tüzer, Deniz Eyice, Pelin Karadağ, Raif Coşkun, Semra Demir, Bahaauddin Çolakoğlu, Suna Büyüköztürk, Aslı Gelincik

**Affiliations:** a Division of Immunology and Allergic Diseases, Şişli Hamidiye Etfal Research and Education Hospital, Istanbul, Turkey; b Division of Immunology and Allergic Diseases, Department of Internal Medicine, Istanbul Faculty of Medicine, Istanbul University, Istanbul, Turkey; c Division of Immunology and Allergic Diseases, Okmeydanı Research and Education Hospital, Istanbul, Turkey

**Keywords:** Anxiety, COVID-19, Omalizumab, Pandemics, Urticaria

## Abstract

**Background:**

The course of chronic spontaneous urticaria (CSU) can be influenced by infections, depression, and stress.

**Objective:**

Our aim was to investigate the impact of the COVID-19 pandemic on the course of refractory CSU together with patient adherence to omalizumab and treatment adjustments.

**Methods:**

Urticaria Activity Score (UAS7) was used to assess disease activity. Fear of COVID-19 Scale (FC-19s), and Depression Anxiety Stress Scale (DASS-21s) were performed to assess mental health status. All scales were performed during the Quarantine Period (QP) and Return to the Normal Period (RTNP). UAS7 Before Pandemic (BP) was recorded from the patients medical records.

**Results:**

The authors evaluated 104 omalizumab-receiving CSU patients. UAS7 scores during QP were significantly higher than those in RTNP and BP (p < 0.01). DASS-21 and FC-19 scores were significantly higher during QP compared to RTNP (p < 0.01). Nineteen (18.2%) patients ceased omalizumab, 9 patients prolonged the intervals between subsequent doses during the pandemic. UAS7 scores in QP were significantly higher in patients who ceased omalizumab than in those who continued (p < 0.001). Among patients who continued omalizumab, 22.4% had an increase in urticaria activity and higher FC-19 scores in comparison with those with stable disease activity (p = 0.008).

**Study limitations:**

The small sample size of patients with prolonged intervals of omalizumab and the lack of mental health evaluation with the same tools prior to the study.

**Conclusion:**

Fear induced by COVID-19 can determine an increase in disease activity. Therefore, patients on omalizumab should continue their treatment and prolonged interval without omalizumab can be considered in patients with good urticaria control.

## Introduction

Chronic Spontaneous Urticaria (CSU) is a debilitating condition presenting with wheals, angioedema, or both lasting for 6 weeks or more.^1^ The international EAACI/GA^2^ LEN/EDF/WAO urticaria guideline recommends second-generation H1-antihistamines as the first-line therapy. Up-dosing to fourfold as second-line therapy can improve response; however, some cases do not respond to antihistamines. Omalizumab is the only approved biological for patients with CSU who remain symptomatic despite a high dose of H1-antihistamine.^1^, ^2^, ^3^, ^4^

The unpredictability of wheals or angioedema and the overwhelming nature of itching can lead to persistent stress and reduced quality of life. Studies have reported an association between stress, anxiety, depression and CSU.^5^, ^6^, ^7^ It is important to assess these factors with validated tools.

Since The World Health Organization declared Coronavirus Disease 2019 (COVID-19) a public health emergency of international concern, a pandemic,^8^ diverse clinical consequences have come to the scene. This unpredictable, fast-spreading infectious disease has been causing universal awareness, but also anxiety and distress.^9^ Since prior studies elucidated that mental well-being had been affected during outbreaks, adverse psychosomatic outcomes are expected to increase.^10^, ^11^, ^12^ The psychological aspect of COVID-19, in terms of fear, stress, anxiety, and depression has yet to be considered thoroughly, especially in patients with chronic diseases.

After the first COVID-19 case was detected in Turkey on March 9, 2020, several containment measures such as social distancing, travel restrictions, and closure of schools and workplaces were implemented. This “Quarantine Period (QP)” lasted for three months. As the number of cases declined by the end of May 2020, restrictions decreased gradually in an effort to maintain routine daily life. This “Return to Normal Period (RTNP)” lasted for three months till the end of August 2020. Behaviors of patients with chronic diseases have been heavily affected during the pandemic considering their adherence to medications, and attendance to follow-up visits however there are no documented data showing the consequences of restrictions on behaviors of patients having chronic diseases. CSU patients can also be expected to be affected similarly by the pandemic and it is important to know the influence of the pandemic on these patients especially when the close relationship between CSU activation and the psychological status of the patients is considered. Therefore, our aim was to investigate the impact of the COVID-19 pandemic on the course of refractory CSU together with patients’ adherence to omalizumab and treatment adjustments.

## Methods

### Study population and the study design

A total of 104 CSU patients who were on omalizumab treatment were recruited at adult outpatient allergy clinics of three tertiary hospitals in Istanbul. Patients who did not attend follow-up visits, who skipped omalizumab injections for various reasons prior to the COVID-19 pandemic, and who were diagnosed as having concomitant psychiatric diseases and malignancies were not included in the study.

Patients were allocated into three groups according to their patterns of omalizumab application. Accordingly, patients who ceased omalizumab (Group 1), patients who extended the time interval between subsequent omalizumab injections (Group 2), and patients who continued regular omalizumab injections (Group 3) during the study period were determined. All subjects were assessed with the study instruments in QP and RTNP. Additionally, urticaria activity data assessed by Urticaria Activity Score (UAS7) Before the Pandemic Period (BP) was obtained from patient's medical records.

### Assessment of urticarial activity and mental status

Disease activity was assessed by UAS7, a simple patient-reported scoring system. Patients assess urticaria by scoring the severity of itch and the number of hives from 0 to 3 over 7 consecutive days.^13^

UAS7 scores were stratified into 5 score bands describing the disease activity from urticaria free to severe urticarial.^14^ Change into a higher-activity disease score band was defined as “increased urticaria activity”.

The mental status of the participants was determined by the Turkish version of the Depression-Anxiety-Stress Scale (DASS-21) and Fear of COVID-19 Scale (FC-19S).^15^, ^16^

The DASS-21 is a 21-item self-reported questionnaire. Respondents indicate how much the statements apply to them over the past week, using a Likert scale ranging from 0 (never) to 3 (most of the time, always).

Fear of COVID-19 was assessed with FCV-19S. The responses of FCV-19S were recorded on a five-point Likert scale ranging from ‘strongly disagree’ (1) to ‘strongly agree’ (5). A higher score represented greater fear of COVID-19.

This study was approved by the Turkish Ministry of Health (TMoH) (2020-09-09T12_40_50) and the institutional review board and the Ethics Committee of the coordinating center (2021‒96399). Informed consent was obtained from all study participants.

### Statistical analysis

Statistical analysis was performed by SPSS.25 version (SPSS Inc., Armonk, NY, USA). Categorical variables were summarized as frequencies and percentages. Continuous variables were given as mean and standard deviations or median (IQR 25‒75). The Wilcoxon test was used for the comparison of data that were not normally distributed. Mann-Whitney *U* test and Kruskal-Wallis test were conducted to evaluate the different groups. The relationship between scores of UAS7, DASS-21, and FC-19 was analyzed by Spearmen’s correlation test. The two-sided p-value <0.05 determined the statistical significance. GraphPad Prism software was used for graphical analysis.

## Results

### Clinical and demographic features of the CSU patients

104 patients were included in the study. The mean age was 42.74 ± 12 years and the majority (74%) of the patients were female. The median duration of CSU was 4 years (IQR 25‒75: 3‒8.75) and the median duration of omalizumab treatment was 24 months (IQR 25‒75: 14‒36.75). Fifty (48.1%) patients had concomitant chronic inducible urticaria as shown in detail in Table 1. Stress was the most common triggering factor (51.9%) whereas infections were triggers in 7.7% of patients. The demographic and clinical features of 104 CSU patients are shown in Table 1.

**Table 1:** Demographic and clinical features of 104 patients.

**Age,** mean years ± SD	42.74 ± 12
**Sex,** n (%)	
Female	77 (74)
Male	27 (26)
**Concomitant disease,** n(%)	46 (44.2)
Cardiovascular Diseases	4 (3.8)
Respiratory Diseases	10 (9.6)
Thyroid diseases	10 (9.6)
Hypertension	10 (9.6)
Arrhythmia	2 (1.9)
Diabetes Mellitus	13 (12.5)
Hyperlipidemia	6 (5.8)
Others	8 (7.7)
**Duration of urticaria,** years, median (IQR 25‒75)	4 (3‒8.75)
**Duration of omalizumab,** months, median (IQR 25‒75)	24 (14‒36.75)
**Presence of angioedema,** n (%)	66 (63.5)
**Presence of inducible urticaria,** n (%)	50 (48.1)
Cold urticaria	13 (12.5)
Solar urticaria	31 (29.8)
Aquagenic urticaria	3 (2.9)
Late pressure urticaria	18(17.3)
Vibratory urticaria	1 (1)
Cholinergic urticaria	13 (12.5)
Contact urticaria	3 (2.9)
Dermographism	23 (22.1)
**Presence of triggering factors,** n (%)	68 (65.4)
Drugs	22 (21.2)
Foods	31 (29.8)
Stress	54 (51.9)
Infection	8 (7.7)
Others	7 (6.7)

### Comparison of the study instruments in all subjects in between pandemic periods

**Figure 1:**
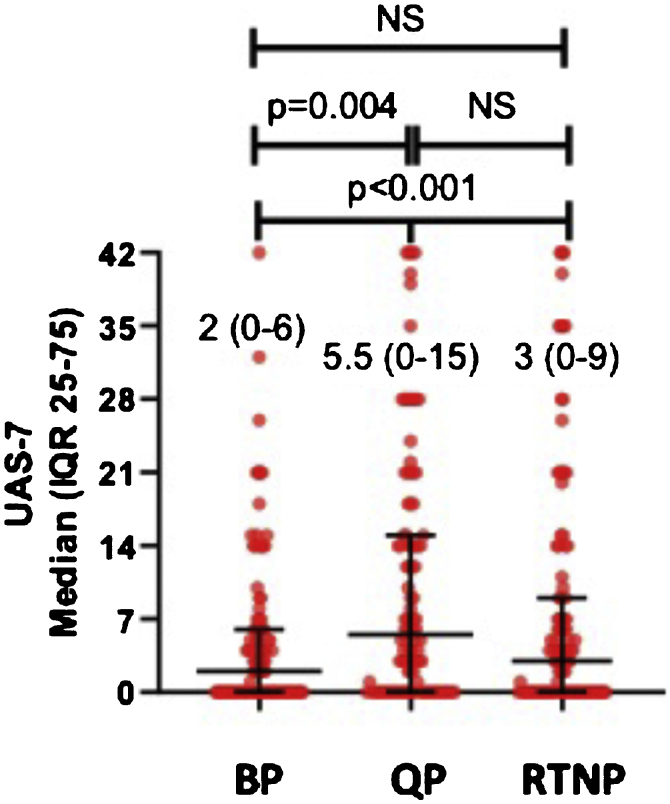
Comparison of median UAS-7 scores in BP, QP, RTNP. UAS-7, Seven Days Urticaria Activity Score; IQR, interquartile range; BP, before pandemic; QP, quarantine period; RTNP, return to normal period; NS, not significant.

UAS7 scores in all patients during QP (UAS7_QP_) were significantly higher than those determined in RTNP (UAS7_RTNP_) and before pandemics (UAS7_BP_) (p < 0.01) In post hoc analysis, median UAS7_QP_ was higher than those determined before pandemics (UAS7_BP_) (p = 0.004), while median UAS7 scores were similar among QP and RTNP (UAS7_RTNP_) (Fig. 1).

**Figure 2:**
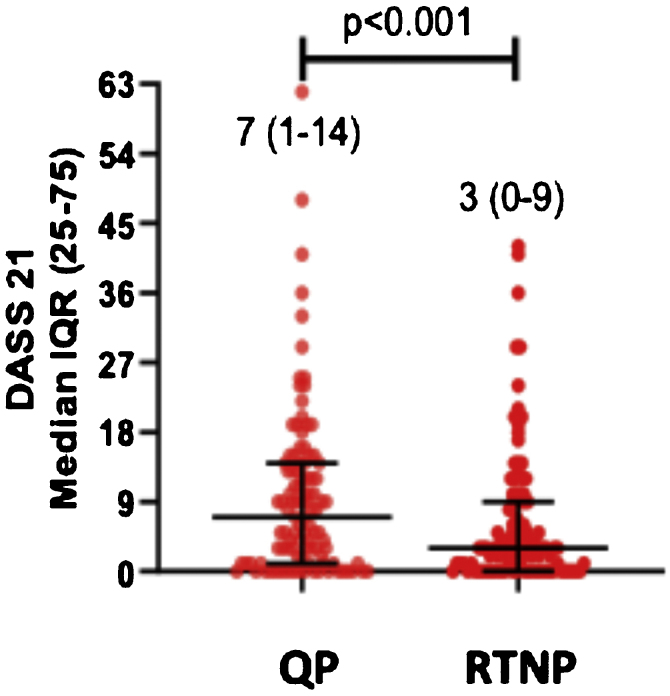
Comparison of median DASS-21 scores in QP and RTNP. DASS-21, Depression Anxiety, and Stress Scale; IQR, interquartile range; QP, quarantine period; RTNP, return to normal period.

**Figure 3:**
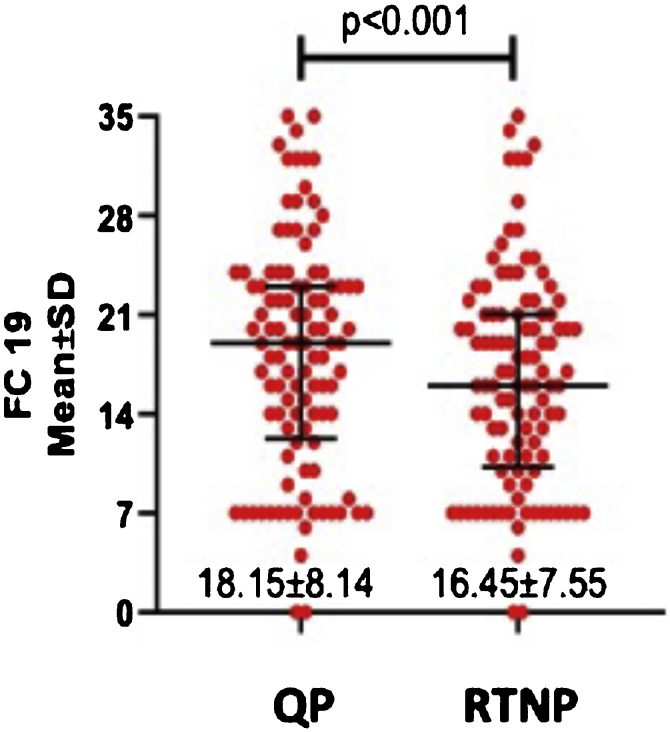
Comparison of mean FC-19 scores in QP and RTNP. FC-19, Fear of COVID-19 scale; QP, quarantine period; RTNP, return to normal period; SD, standart deviation.

DASS-21 and FC-19 scores were significantly higher during QP compared to RTNP (p < 0.01) (Figure 2, Figure 3).

Females had significantly higher scores of FC-19 (p = 0.024) during QP, while UAS7 and DASS-21 didn’t differ between genders. UAS7, DASS-21, and FC-19 scores didn’t differ in terms of age, education status, or presence of angioedema or chronic disease during the two periods (Table 2).

**Table 2:** UAS7, DASS-21s, FC-19s of patients according to demographic and clinical features.

	UAS7 median (IQR: 25‒75)			FC-19 (mean SD)		DASS-21t median (IQR: 25‒75)	
	BP	QP	RTNP	QP	RTNP	QP	RTNP
**Sex**							
Female	2 (0‒5.5)	5 (0‒18)	3 (0‒12)	19.25 ± 7.93	17.31 ± 7.19	7 (2.5‒14.5)	3 (1‒9)
Male	3 (0‒8)	6 (0‒12)	4 (0‒9)	15.04 ± 8.07	14 ± 8.15	6 (0‒14)	1 (0‒9)
p	NS	NS	NS	0.024	NS	NS	NS
**Age**							
18‒40	2 (0‒5.25)	4.5 (0‒14.25)	3 (0‒7.5)	18.45 ± 7.66	16.64 ± 6.49	8 (1‒14)	3 (0‒10.5)
40‒65	4 (0‒8.5)	7 (0‒19.5)	4 (0‒12.5)	18.34 ± 8.52	16.7 ± 8.29	7 (1.5‒13.5)	3 (1‒9)
> 65	0 (0‒0)	0 (0‒21)	0 (0‒15)	14 ± 5.09	11.5 ± 3.11	3.5 (0‒9.25)	3 (0‒9)
p	NS	NS	NS	NS	NS	NS	NS
**Presence of angioedema**	2 (0‒6)	5 (0‒15)	3 (0‒7.5)	18.94 ± 8.13	17.09 ± 7.66	9 (3‒14.25)	3.5 (1‒10)
p	NS	NS	NS	NS	NS	NS	NS
**Presence of chronic diseases**	1 (0‒7)	10.5 (0‒21.75)	4.5 (0‒14)	18.67 ± 8.44	16.67 ± 8.02	6 (1‒11)	3 (0‒8.25)
p	NS	NS	NS	NS	NS	NS	NS

UAS-7, Seven Days Urticaria Activity Score; DASS-21, Depression Anxiety and Stress Scale; FC-19, Fear of COVID-19 scale; IQR, Interquartile Range; SD, Standart Deviation; BP, Before Pandemic; QP, Quarantine Period; RTNP, Return to Normal Period; NS, Not Significant.

**Figure 4:**
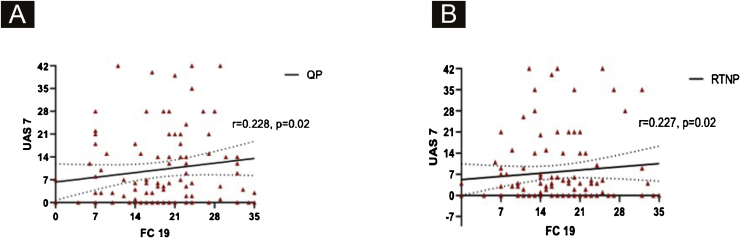
(A) Correlation of scores of UAS7 and FC-19s in QP. (B) Correlation of scores of UAS7 and FC-19s in RTNP. UAS-7, Seven days Urticaria Activity Score; FC-19, Fear of COVID-19 scale; QP, quarantine period; RTNP, return to normal period.

**Figure 5:**
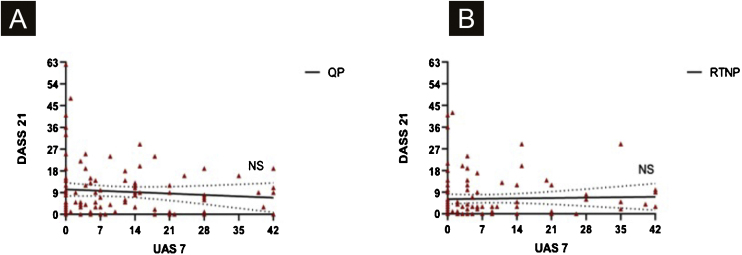
(A) Correlation of scores of DASS-21 and UAS7 in QP. (B) Correlation of scores of DASS-21 and UAS7 in RTNP. DASS-21, Depression anxiety and stress scale; UAS-7, Seven days urticaria activity score; QP, quarantine period; RTNP; return to normal period.

UAS7 scores and FC-19 scores were correlated to each other during QP and RTNP (p = 0.02, R = 0.228, p = 0.02, R = 0.227 respectively) (Fig. 4 A‒B), while UAS7 scores were not correlated to DASS-21 scores. (Fig. 5 A‒B).

**Figure 6:**
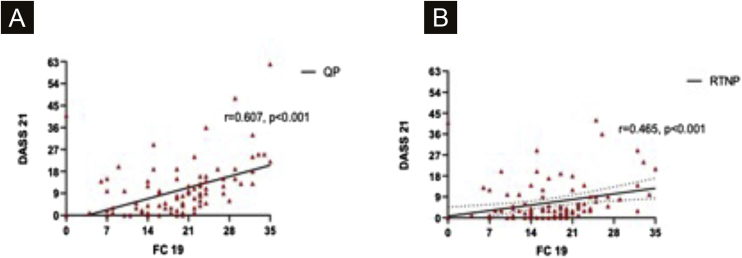
(A) Correlation of scores of DASS-21 and FC-19s in QP. (B) Correlation of scores of DASS-21 and FC-19s in RTNP. DASS-21: Depression anxiety and stress scale, FC-19: Fear of Covid-19 scale, QP: quarantine period, RTNP: return to normal period.

DASS-21 and FC-19 were correlated to each other during QP and RTNP (p < 0.001, R = 0.607, p = 0.02, R = 0.465 respectively) (Fig. 6 A‒B).

### Determination of study groups according to omalizumab application during the pandemic

Seventy six patients had omalizumab injections with the same dose intervals (Group 3). Injection intervals were prolonged in 9 patients (Group 2) and 19 (18.3%) patients ceased omalizumab (Group 1). The reason for prolongation of omalizumab intervals was fear of COVID-19, while the most common reasons for cessation of omalizumab were fear of COVID-19 and difficulty to attend to hospitals. Some patients had more than one reason for cessation of omalizumab during QP (Table 3). 20% of patients in Group 3, had their injections at another center different from their initial center, 2.4% had home therapy and the rest continued at their initial center during the study period. In Group 2, the median duration of intervals between omalizumab doses was 8 weeks (IQR 25‒75: 6‒8). In Group 1, the median duration of omalizumab cessation was 16 weeks (IQR 25‒75: 14‒19).

**Table 3:** The replies regarding the reasons for the ceasation of omalizumab during the quarantine period of the patients.

	Number of patients (%)
I didn’t attend a healthcare institution due to fear of COVİD-19.	12 (63.1)
I had difficulty finding appointments and/or transportation to hospitals.	6 (31.5)
I didn’t know facilitation of access to prescribed medication during the pandemic.	5 (26.3)

### Comparison of urticarial activity among patient groups before the pandemic

Patients in Group 3 had higher mean UAS7_BP_ than those in Group 1 or the ones in Group 2 (p = 0.021). Post hoc analysis revealed that the median UAS7_BP_ was higher in Group 3 than those in Group 2 (p = 0.015), while the median UAS7_BP_ was similar between patients in Group 3 and those in Group 1 (Table 4).

**Table 4:** UAS7, DASS-21s, FC-19s scores of 3 patient groups in BP, QP and RTNP.

		Group 1 (19 patients)	Group 2 (9 patients)	Group 3 (76 patients)	p
**UAS-7 median (IQR: 25‒75)**	BP	0 (0‒6)	0 (0‒1)	3 (0‒7)	0.021
	QP	28 (12‒39)	0 (0‒6,5)	4 (0‒14)	<0.001
	RTNP	0 (0‒26)	1 (0‒21)	4 (0‒9)	NS
**FC-19s (mean ± SD)**	QP	19.42 ± 6.69	16.89 ± 9.29	17.99 ± 8.4	NS
	RTNP	16.47 ± 6.12	14.78 ± 7.44	16.64 ± 7	NS
**DASS-21s median (IQR: 25‒75)**	QP	9 (0‒15)	6 (0.5‒12.5)	5.5(1‒14)	NS
	RTNP	4 (0‒12)	2 (0‒4.5)	3 (0‒9)	NS

UAS-7, Seven Days Urticaria Activity Score; FC-19, Fear of COVID-19 scale; DASS-21, Depression Anxiety and Stress Scale; IQR, Interquartile Range; SD, Standart Deviation; BP, Before Pandemic; QP, Quarantine Period; RTNP, Return to Normal Period; NS, Not Significant.

### Comparison of study instrument results among patient groups in two pandemic periods

In QP, the median UAS7 was different among the three groups (p < 0.001). In post-hoc analysis, the median UAS7_QP_ was the highest in patients in Group 1 (p < 0.001), while the median UAS7_QP_ was similar between patients in Group 2 and those in Group 3 (p > 0.05) (Table 4).

In all three groups, the median UAS7_QP_ was higher than the median UAS7_BP_, and this increase was statistically significant in patients in Group 1 (p < 0.001) and in those in Group 3 (p = 0.008).

FC-19 scores and DASS-21t scores didn’t differ among the three groups in QP (Table 4).

In RTNP, it was observed that all Group 1 patients restarted omalizumab since their symptoms deteriorated. The median UAS7, FC-19, and DASS-21 scores were not different among the three groups in RTNP (Table 4).

### FC-19 scores in patients with increased urticaria activity during QP

Thirty four (32.7%) patients had increased urticaria activity during QP. Among these patients, FC-19 scores during QP and having comorbidities that are associated with more severe COVID-19 were statistically higher than those with stable urticarial activity (p = 0.013, p = 0.03, respectively). 22.4% of patients in Group 3 had an increase in urticaria activity during QP and these patients had higher FC-19 scores in comparison with those with stable disease activity (p = 0.008).

## Discussion

This is the first study investigating the impact of the COVID-19 pandemic on refractory CSU patients treated with omalizumab. Our findings revealed that the COVID-19 pandemic can impair the course of refractory CSU and interventions to support ongoing treatments are therefore crucial. Furthermore, it provides an additional contribution to the recommendations of the recent EAACI statement on biological treatment in CSU during the pandemic^17^ by showing that up to 8 weeks extension in subsequent omalizumab injections can be tolerated in refractory CSU during the pandemic.

CSU patients are shown to have higher levels of stress and disease activity is correlated with emotional stress levels.^18^, ^19^ In a recent study evaluating the impact of the pandemic on 509 mild/moderate CSU patients, revealed increased urticaria activity and higher stress levels during QP compared to BP and RTNP.^20^ In this present study, the authors were able to determine the increase in disease activity in refractory CSU patients receiving omalizumab during the pandemic. Since emotional disturbances and stressful life events influence CSU courses, it might be speculated that the COVID-19 pandemic would have detrimental effects on CSU patients.

Fear is one of the characteristic features of infectious diseases.^21^ With the high infection rate and relatively high mortality, individuals can worry about COVID-19. In this respect, the authors used the FC-19 scale which is a valid tool for assessing fear of COVID-19 and significantly correlated with depression and anxiety.^16^ Shin et al.^22^ also stated that the fear/anxiety neurocircuitry has overlap and interacts with the neurocircuitry that orchestrates the stress response. In our study, FC-19 scores were significantly higher during QP compared to RTNP, and FC-19 scores were correlated to DASS-21 scores. Furthermore, among patients who continued omalizumab treatment, those showing an increase in urticaria activity had higher FC-19 scores in QP, indicating the impact of fear on the course of CSU.

Response to a psychosocial stressor is different between genders. Reduced fear conditioning is seen in stressed men whereas stressed women can show enhanced fear learning.^23^, ^24^ This might be the probable explanation for our female patients displaying higher FC-19 scores while DASS-21 scores weren’t different than men.

The containment measurements such as reducing visits to hospitals and isolation at home implemented to avoid the spread of this highly contagious disease became an obstacle for follow-up and treatment of chronic diseases. Similarly, Kocatürk et al. stated that the weekly number of CU patients treated at UCARE centers decreased by more than 50% during the COVID-19 pandemic.^25^

A study evaluating the effects of the pandemic on CU patients revealed 44% of omalizumab cessation.^26^ In our study, 81.7% of the patients continued omalizumab. This relatively higher rate of adherence can be attributed to the regulations in our clinics for the continuation of ongoing omalizumab treatments during the pandemic.

Omalizumab can only be injected in hospitals via prescription in Turkey. During QP, TMoH facilitated access to prescribed medication for those who had valid prescription consent reports. By the courtesy of access to prescribed medication, 20% of our patients continued their injections at different centers other than their initial healthcare center, and 2.4% of the patients had home therapy. At this point, the authors should emphasize the importance of specific attention to tailored interventions in medications for chronic diseases during special periods such as outbreaks in order to maintain effective follow-up and treatment. A recent study by Öztürk et al. stated that 59% of the adult/pediatric allergists in Turkey used telemedicine for the management of urticaria/angioedema patients.^27^

The recent EAACI statement on the usage of biologicals during COVID-19 advised not to change therapy in non-infected individuals based on expert opinion.^17^ Nevertheless, successful omalizumab treatment adjustments have been shown in patients with refractory CSU before the pandemic.^28^, ^29^, ^30^ In our study, the authors for the first time showed that dose interval adjustments can be tolerated in refractory CSU patients during the pandemic, but cessation can worsen the course of the disease. Patients those prolonged omalizumab intervals had higher UAS7_BP_ scores. Therefore, interval prolongation can be recommended for patients with good urticaria control in order to reduce hospital visits during the pandemic.

On the other hand, 78.9% of patients who ceased omalizumab had increased urticaria activity. Since our patients were refractory CSU patients, loss of disease control when omalizumab was halted was expected.

COVID-19 has been a risk for all human beings and the presence of comorbidities such as diabetes mellitus, hypertension, cardiovascular disease, and chronic respiratory disease were identified as risk factors for severe COVID-19. In our study, patients having these comorbidities had higher UAS7, DASS-21t, and FC-19s, but weren’t statistically significant. Nevertheless, 47.1% of them had an increase in urticaria activity during QP and this is statistically significant when compared to patients without comorbidities. It can be speculated that knowing to have comorbidities that are associated with severe COVID-19 infection resulted in the loss of disease control, which might be another indicator of the association of fear and stress with urticaria activity.

There were two limitations in this study. The first is the small sample size of patients with prolonged intervals of omalizumab which may have affected the accuracy of statistical results. The authors recommended our patients not change therapy according to the EAACI statement.^17^ Patients in Group 2 prolonged the intervals independent of our suggestions.

As the second limitation, the authors could not evaluate the mental health of our patients with the same tools prior to the study.

## Conclusion

The authors found that both the fear and stress induced by COVID-19 and cessation of omalizumab yielded an increase in urticaria activity. Therefore, patients on omalizumab should continue their treatment either with the same dose intervals or prolonged intervals and tailored interventions such as telemedicine and home therapy should be considered for patients with chronic diseases during periods of outbreaks in order to maintain effective follow-up and reduce unnecessary visits to hospitals.

## Financial support

None declared.

## Authors' contributions

Müge Olgaç: Has made substantial contributions to conception and design, acquisition of data, analysis, and interpretation of data; has been involved in drafting the manuscript, revising it critically for important intellectual content.

Osman Ozan Yeğit: Has made substantial contributions to or acquisition of data, analysis and interpretation of data; has been involved in drafting the manuscript, revising it critically for important intellectual content.

Sengul Beyaz: Has made substantial contributions to the acquisition of data, analysis and interpretation of data; has been involved in drafting the manuscript.

Özdemir Can Tüzer: Has made substantial contributions to the acquisition of data, analysis and interpretation of data; has been involved in drafting the manuscript.

Deniz Eyice Karabacak: Has made substantial contributions to conception and design, acquisition of data, and has been involved in drafting the manuscript.

Nida Oztop: Has made substantial contributions to the acquisition of data, analysis and interpretation of data; has been involved in drafting the manuscript.

Pelin Karadağ: Has made substantial contributions to the acquisition of data, and interpretation of data; has been involved in drafting the manuscript.

Raif Coşkun: Has made substantial contributions to conception and design, acquisition of data, or analysis and interpretation of data; has been involved in drafting the manuscript.

Semra Demir: Has made substantial contributions to conception and design, acquisition of data, analysis and interpretation of data; has been involved in drafting the manuscript and revising it critically for important intellectual content.

Bahauddin Colakoglu: Has made substantial contributions to conception and design, acquisition of data, analysis and interpretation of data; has been involved in drafting the manuscript or revising it critically for important intellectual content.

Suna Buyukozturk: Has made substantial contributions to conception and design, acquisition of data, analysis and interpretation of data; has been involved in revising the manuscript critically for important intellectual content.

Aslı Gelincik: Has made substantial contributions to conception and design, acquisition of data, analysis and interpretation of data; has been involved in revising the manuscript critically for important intellectual content.

## Conflicts of interest

None declared.
